# Lysine methylation of FEN1 by SET7 is essential for its cellular response to replicative stress

**DOI:** 10.18632/oncotarget.18070

**Published:** 2017-05-22

**Authors:** Palaniraja Thandapani, Anthony M. Couturier, Zhenbao Yu, Xing Li, Jean-François Couture, Shawn Li, Jean-Yves Masson, Stéphane Richard

**Affiliations:** ^1^ Terry Fox Molecular Oncology Group and Bloomfield Center for Research on Aging, Lady Davis Institute for Medical Research, Jewish General Hospital, Montréal, Québec, Canada; ^2^ Departments of Oncology and Medicine, McGill University, Montréal, Québec, Canada; ^3^ Genome Stability Laboratory, Laval University Cancer Research Center, CRCHU de Québec, Québec, Canada; ^4^ Department of Biochemistry, Schulich School of Medicine and Dentistry, Western University, London, Ontario, Canada; ^5^ Ottawa Institute of Systems Biology, Department of Biochemistry, Microbiology, and Immunology, University of Ottawa, Ottawa, Ontario, Canada

**Keywords:** lysine methylation, SET7, FEN1, DNA damage response, DNA replication

## Abstract

The DNA damage response (DDR) is central to the cell survival and it requires post-translational modifications, in part, to sense the damage, amplify the signaling response and recruit and regulate DNA repair enzymes. Lysine methylation of histones such as H4K20 and non-histone proteins including p53 has been shown to be essential for the mounting of the DDR. It is well-known that the lysine methyltransferase SET7 regulates the DDR, as cells lacking this enzyme are hypersensitive to chemotherapeutic drugs. To define addition substrates of SET7 involved in the DDR, we screened a peptide array encompassing potential lysine methylation sites from >100 key DDR proteins and identified peptides from 58 proteins to be lysine methylated defining a methylation consensus sequence of [S>K^-2^; S>R^-1^; K^0^] consistent with previous findings. We focused on K377 methylation of the Flap endonuclease 1 (FEN1), a structure specific endonuclease with important functions in Okazaki fragment processing during DNA replication as a substrate of SET7. FEN1 was monomethylated by SET7 *in vivo* in a cell cycle dependent manner with levels increasing as cells progressed through S phase and decreasing as they exited S phase, as detected using K377me1 specific antibodies. Although K377me1 did not affect the enzymatic activity of FEN1, it was required for the cellular response to replicative stress by FEN1. These finding define FEN1 as a new substrate of SET7 required for the DDR.

## INTRODUCTION

The DNA damage response (DDR) pathway consists of a complex cellular response implicating a plethora of factors responsible for detection, signaling and repair of the DNA breaks [1]. The initial response to DNA double strand breaks (DSBs) requires the orderly recruitment of many proteins including protein kinases, ubiquitin and SUMO E3 ligases and adaptor molecules that will mediate the signaling of DNA damage and its repair [2, 3]. Arginine and lysine methylation have been shown to also be key regulators the DDR [4-6]. Arginine methylation of MRE11 by PRMT1 regulates the function of MRE11 during DDR and is essential for maintenance of genomic stability [7, 8]. The list of DNA damage proteins known to be arginine methylation now include 53BP1, p53, FEN1, BRCA1, RAD9, DNA polymerase β are known to be arginine methylated (reviewed in [4, 6, 9]. Much more is known about lysine methylation as there are many more lysine methyltransferases, lysine demethylases and the methylmarks are recognized by several readers with Tudor, MBT, PWWP chromo and PHD domains.

Lysine methylation of histones plays key roles in the transcriptional regulation and the DNA damage response. H4K20 is required for the requirement of the 53BP1 at DNA double stranded breaks in higher eukaryotes. H4K20 methylation is mediated by SETD8 and SUV4-20H1/2 (for review [10, 11]). Methylation of non-histone also play key roles. The methylation of p53 by SMYD2, G9a/GLP and SET8 are linked to suppression of p53 function [12-14]. Another lysine methyltransferase (KMT7) also known as SET (Su(var)-3-9, Enhancer-of-Zeste, Trithorax)7, SET9, and SET7/9 catalyzes the monomethylation of free histones, but not nucleosomes, has been shown to regulate the DDR. Peptide SPOT arrays have been used to define the substrate specificity of SET7 and the following consensus target motif [K>R^-2^; S>KYARTPN^-1^;K^0^;QN^+1^;AQGMSPTYV^+2^] was identified [15]. This consensus sequence expanded the previously proposed SET7/9 methylation site [K/R^-2^;S/T/A^-1^;K^0^] defined by crystallographic studies [16]. Numerous non-histone substrates have been identified for SET7 including TAF10, ERα, DNMT1, RelA, STAT3, YY1, Gli3 and Yap [17-23]. SET7 plays multiple roles in the DNA damage response pathway, as cells deficient for SET7 are hypersensitive to agents that induce DNA damage including doxorubicin [24]. A role for SET7 in DDR was first highlighted when it was shown to methylate p53 [25]. SET7 methylation of p53 post-DNA damage induced cell cycle arrest by stabilizing p53 and increasing p21 expression. SET7 methylation of RB and E2F-1 was identified to regulate RB dependent cell cycle arrest and apoptotic functions of E2F-1, respectively [26, 27]. In addition, Ivanov et al., (2007) reported an increase in the enzymatic activity of SET7 following treatment with DNA damage agent adriamycin, suggesting that SET7 could methylate several substrates involved in the DDR pathway [28]. In the present study, we used an *in vitro* peptide SPOT array to identify and characterize new substrates of SET7 in the DDR pathway. We identified many DDR proteins including the Flap endonuclease I (FEN1) to be *in vitro* methylated by SET7.

FEN1 is a structure-specific endonuclease that functions in the excision of Flap structures that arise from Okazaki fragment maturation during lagging strand synthesis and long patch base excision repair [29, 30]. In addition, FEN1 possesses 5′-exonuclease and gap-endonuclease activities. These distinct nuclease activities have allowed FEN1 to participate in multiple DNA repair events like resolution of stalled replication forks, maintenance of telomere stability and prevention of tri-nucleotide repeat expansion [31]. In this study, we report a new mechanism of regulation of FEN1 function by SET7 methylation. We show that FEN1 is monomethylated by SET7 *in vitro* and *in vivo* on lysine 377 (K377me1). We further show that K377me1 is upregulated during S phase progression in a SET7-dependent manner. In addition, we identify FEN1K337me1 is required for the cellular response to hydroxyurea (HU).

## RESULTS

### FEN1 is an *in vitro* substrate of SET7

**Figure 1:**
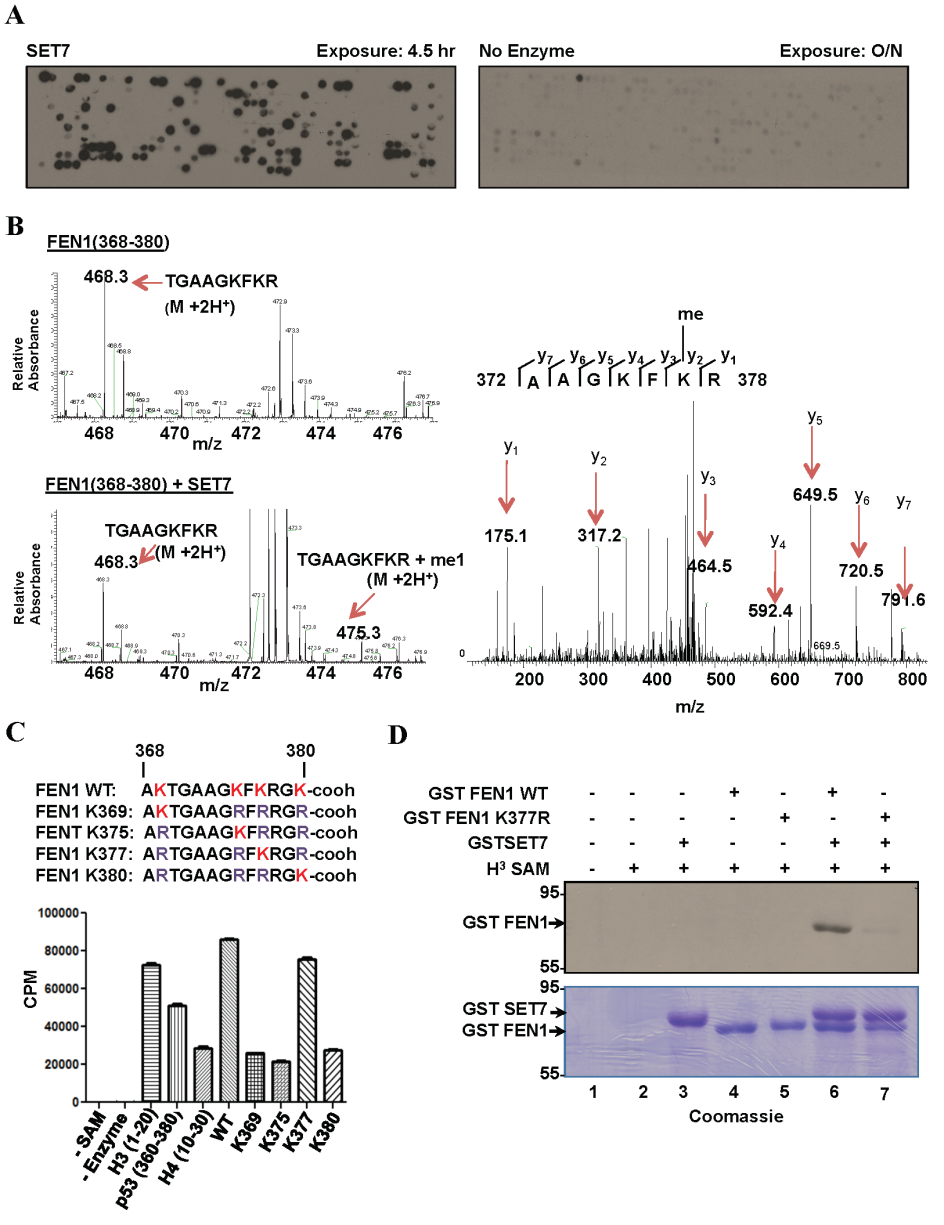
SET7 methylates FEN1 at K377 **A.** Peptide array encompassing lysine methylation sites as predicted by the software MEMO from over 100 DNA damage response proteins were synthesized on a membrane which was incubated with SET7 (left) or no enzyme (right) in the presence of H_3_-Sadenosyl-L-methionine as the methyl donor. The methylated peptides were visualized by fluorography. **B.** Mass spectrometry analysis of methylation assays of FEN1 peptide (amino acid 368-380); MS spectra of FEN1 (368-380) without (top) and with (bottom) SET7. MS/MS spectra of the monomethylated peptide fragment (right). **C.** In vitro methylation assays were carried out using biotinylated peptide substrates harbouring K to R mutations of FEN1 (368-380). H_3_-S-adenosyl-L-methionine was used as the methyl donor. Methylation was carried out in Streptavidin Flashplate and analyzed by scintillation counter. Error bars represent SD, (*n* = 3). **D.** Methylation assays were performed on full length recombinant GST FEN1 WT and GST FEN1 K377R. The proteins were resolved by SDS-PAGE, stained with Coomassie blue (bottom) dried and analyzed by fluorography (top).

To identify other DDR substrates of SET7, we synthesized a peptide SPOT array encompassing 461 potential lysine methylation sites, as predicted by the protein methylation prediction tool MeMo, from 118 proteins known to play a role in the DNA damage response or DNA repair (Supplementary Table 1). The peptide array was incubated with recombinant SET7 in the presence of ^3^H-methyl-*S*-adenosyl-L-methionine and methylation was visualized by fluorography. We identified 121 peptides that were methylated by SET7 (Figure 1A, Supplementary Table 2). We identified peptides from XPC, XPD, XPF, XPG, CSB, FEN1, ATR, ATM, ATRIP, DNA-PK, 53BP1, BARD1, BAP1, BRCA2, FANCB, FANCD2, FANCM, RAD51, RAD51C, RAD50, NBS1, RNF168, H2AX, CHK1, RAD18, TOPBP1, Claspin, Timeless, TIP60, BLM, WRN, RPA1, MLH1, MLH3, MSH2, MSH6, EXO1, LIG1, LIG3, LIG4, POLZ, REV1, XRCC1, XRCC3, APEX1, MBD4, NTHL1, NEIL1, NEIL2, PARP1, PARP2, PARP3, APC, p53, p73, ING2, p107, MCM3, and MCM8 to be substrates for SET7. Over 300 peptides were not methylated including peptides from SMC1, BRCA1, USP7, MRE11, MDC1, and RAP80, demonstrating that SET7 does not methylate any lysine residue containing peptide. Curating the data from the 121 methylated peptides revealed the selected sequences [26S>15K>12A/11L/11G^-2^; 27S>15R>12L/11A^-1^; K0; 31K>19S>13E/13G^+1^; 20K/14T/13G^+2^] defining a consensus [S>K^-2^; S>R^-1^; K0] with lysine being selected at the +1 and +2 positions. Therefore, our peptides fit the previously identified consensus sequence with K^-2^ and S^-1^ [15, 16], and reveal that SET7 can contain serine or a small hydrophobic amino acid (A/L/G) at the -2 position. Furthermore, R and L/A were also preferred at the -1 position.

Considering the divergence, especially in regard to the residues in position -1 and +1, between the FEN1 methylation site and SET7/9 consensus site, we performed molecular modeling of the FEN1 peptide in SET7/9 SET domain. Starting from the structure of SET7/9 bound to histone H3, we modeled the FEN1 peptide and performed geometry refinement and manual adjustment of the endonuclease’s amino acids modeled in proximity of the substrate. As shown in Supplementary Figure 1, the peptide, which include residues ^375^GKFKRGK^380^, is modeled in a U-shape conformation with G375 and K380 protruding out SET7/9 binding cleft. In FEN1, K375 is modeled within a cleft composed of residues Asp256 and Trp260 and adopt a similar orientation as previously observed in the crystal structure of SET7/9 bound to p53 [25]. In position -1, F376 is modeled in a pocket composed of the FEN1 backbone (residues 379 and 380), Val274 and the aliphatic portion of His252 and Ser268. In the FEN1 peptide, the position +1 is occupied by an arginine and is modeled in close proximity of Asp306. Finally, in the model, G379 and K380 exit the peptide binding cleft and are found in proximity of the N-terminus of the peptide. Interestingly K380 is the last residue of FEN1 and therefore a carboxylate was added to the C-terminus of the peptide. Overall, our modeling studies further support that FEN1 is a *bona fide* substrate for SET7/9.

From our peptide array, we identified peptides (amino acids 354-368; KRKEPEPKGSTKKKA; 368-380; AKTGAAGKFKRGK) covering the C-terminal region of FEN1 to be methylated by SET7. We selected the DNA Flap Endonuclease 1, FEN1, for further investigation, as it deviates from the known consensus [K^-2^S^-1^K^0^] with its methylation sites being EPKGS or AGKFK/ KFKRG. In addition, FEN1 has been shown to be phosphorylated, acetylated, arginine methylated, ubiquinylated and sumoylated, but not lysine methylated [32]. We focused our attention on the C-terminal peptide, as it is the site of acetylation by p300 at K375, K377 and K380 [33]. First to identify the specific lysine residue targeted by SET7, a peptide covering amino acids 368-380 of FEN1 was *in vitro* methylated by SET7 and analyzed by mass spectrometry. SET7 incubation resulted in the detection of a monomethylated lysine residue consistent with SET7 catalyzing monomethyllysines [34, 35]. Subsequent mass spectrometry on the monomethylated peptide identified K377 as the site methylated by SET7 (Figure 1B). To confirm K377 as the primary site of SET7 methylation, biotinylated peptides, harbouring K to R mutations of the FEN1 (368-380) sequence were synthesized. *In vitro* methylation assay on these peptides identified K377 of FEN1 as the site methylated by SET7 (Figure 1C). *In vitro* methylation on full length FEN1 and the K377R mutant was carried out. SET7 was able to methylate full length FEN1, but not the K377R mutant (Figure 1D). This further validated K377 as the primary site of SET7 methylation in full length FEN1.

### *In vivo* methylation of FEN1 by SET7

**Figure 2:**
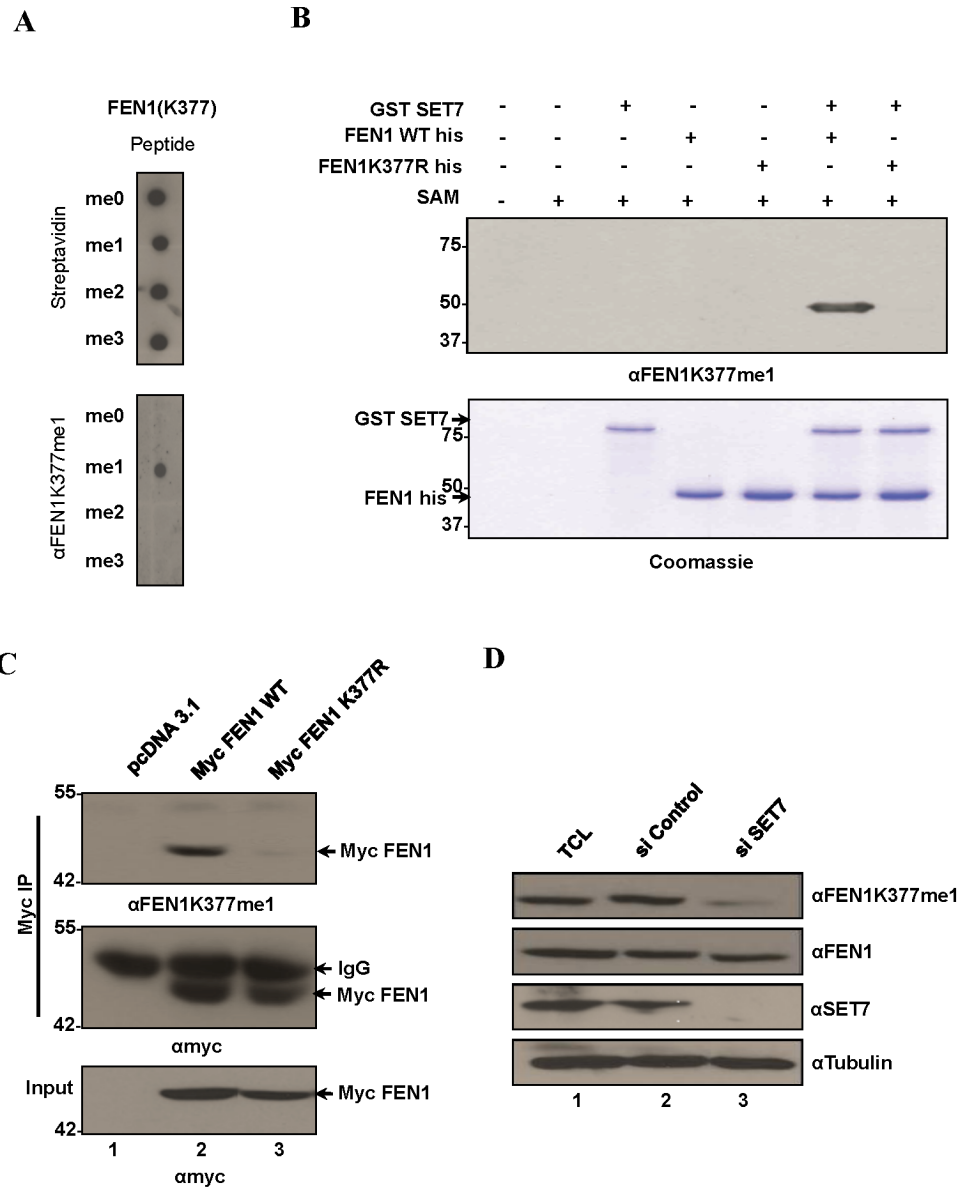
FEN1 is monomethylated at K377 by SET7 *in vivo* **A.** Specific recognition of FEN1K377me1 by the FEN1 K377 monomethyl-specific antibody (αFEN1K377me1). Equal amounts of biotinylated K377 unmodified, mono-, di and tri methylated FEN1 peptides were immunoblotted with αFEN1K377me1. Blots were probed with HRP-conjugated streptavidin to control for equal loading. **B.** αFEN1K377me1 antibody recognizes FEN1 in vitro methylated at K377 by SET7. Immunoblot analysis of recombinant FEN1WT protein or K377R mutant methylated by SET7. **C.** FEN1 is methylated endogenously at K377. Western blot analysis of myc immunoprecipitates from U2OS whole-cell extracts expressing Myc FEN1 WT and the K377R mutant. αFEN1K377me1 antibody recognized immunoprecipitated WT FEN1 but not the K377R mutant. Western blot with anti-Myc served as loading controls. **D.** Knockdown of endogenous SET7 in U2OS cells decreases endogenous levels of K377me1 as detected by immunoblotting with αFEN1K377me1. Western blots with FEN1, SET7 and tubulin are shown.

To determine the *in vivo* methylation of FEN1 at K377, we raised an antibody against the FEN1K377me1 epitope (referred to as anti-FEN1K377me1 hereafter). Anti-FEN1K377me1 antibodies specifically recognized FEN1K377me1 peptides and did not cross-react with unmodified FEN1 (me0), FEN1K377me2 or FEN1K377me3 peptides (Figure 2A). Moreover, anti-FEN1K337me1 recognized recombinant His tagged FEN1 WT incubated with GST-SET7, but not the K377R mutant (Figure 2B). Based on these results, we conclude that anti-FEN1K377me1 antibodies are specific to the K377me1 form of FEN1 and could be utilized to monitor methylation of this site *in vivo*. To determine whether FEN1 is methylated at K377 *in vivo*, U2OS cells were transfected with myc-epitope tagged wild type FEN1 and myc-FEN1 harboring an amino acid substitution K377 to R (myc-FEN1:K377R). Immunoblot analysis of the anti-myc immunoprecipitates with the anti-FEN1K377me1 antibodies revealed the methylation of the wild type FEN1, but not FEN1:K377R (Figure 2C), suggesting that FEN1 is monomethylated at K377 in U2OS. We next investigated whether SET7 is the physiological enzyme responsible for the monomethylation of FEN1K377. We depleted SET7 expression in U2OS cells using SMARTPOOL siRNAs and monitored the methylation of FEN1K377. Immunoblotting of lysates from control and SET7-depleted cells showed reduction of FEN1K377me1 in SET7 siRNA-treated cells (Figure 2D). These findings show that SET7 is the physiological enzyme methylating FEN1 at K377 *in vivo*.

### K377me1 is regulated by SET7 during S phase

To define a functional role for K377me1, we investigated whether it is regulated in response to DNA damage and/or during the phases of the cell cycle. FEN1 has been shown to be acetylated by the histone acetyltransferase p300 in response to UV light on C terminal lysines K354, K375, K377 and K380 [33]. Since methylation and acetylation of a lysine residue are mutually exclusive events, we investigated whether FEN1K377me1 is downregulated in response to UV radiation. U2OS cells were treated with UV radiation and the cellular lysates were collected at various time points and immunoblotted with FEN1K377me1 antibody. FEN1K377me1 was not regulated in response to UV damage, whereas γH2AX, a known marker of DNA damage accumulated after UV damage (Supplementary Figure 2A). K377me1 levels were also not affected in response to DNA damage treatment with methylmethanesulfonate (MMS, Supplementary Figure 2B).

**Figure 3:**
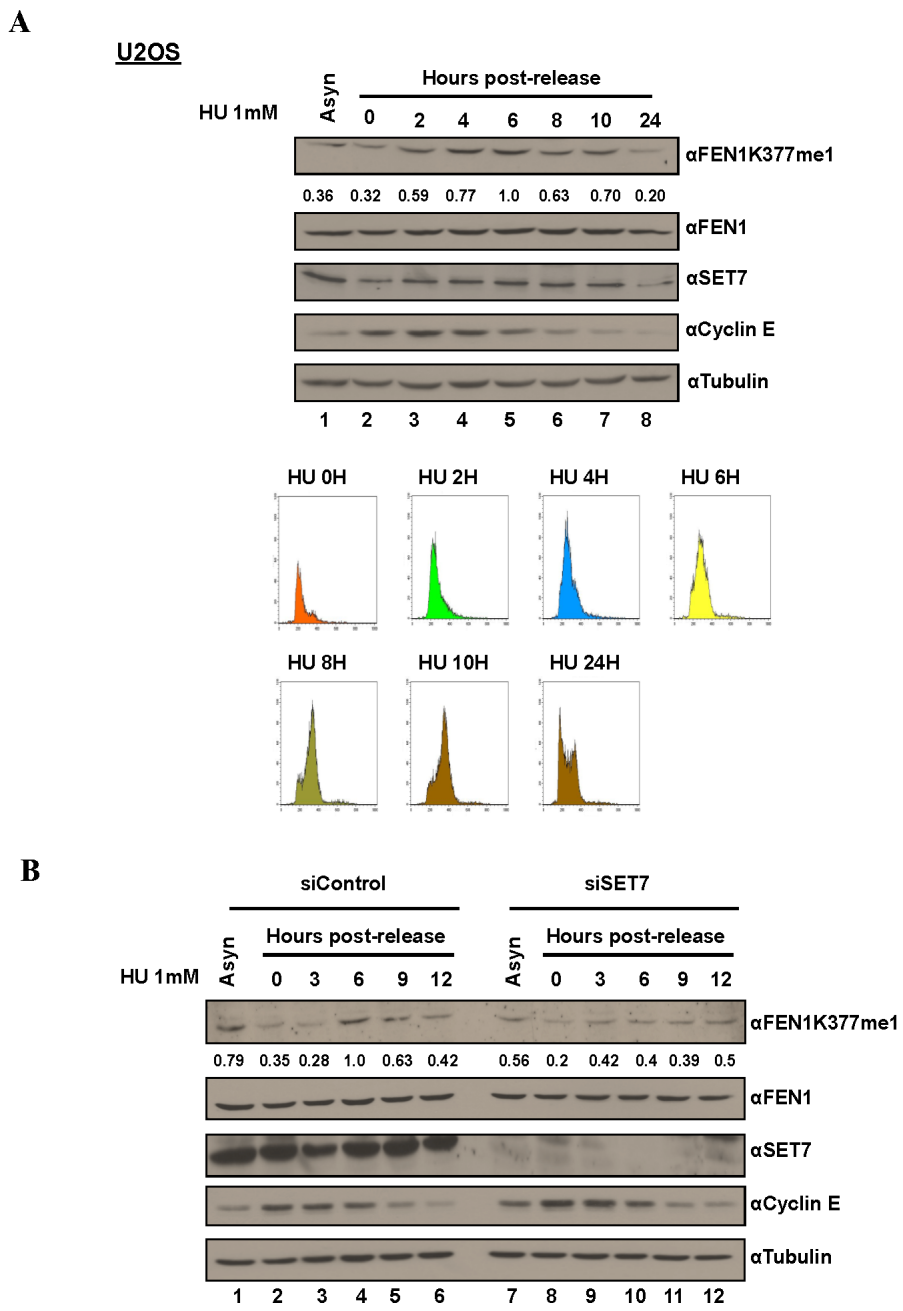
FEN1K377me1 is regulated as U2OS cells progress through S phase **A.** Proteins extracted from the synchronized cells were immunoblotted with the antibodies specific against the indicated proteins. FENK377me1 levels normalized to total FEN1 show the increase of FEN1K377me1 as cells progress through S phase. FACS analysis of U2OS cells showing the synchronization of cells by HU and the subsequent release (bottom). **B.** Knockdown of SET7, prior to synchronisation with HU results in the loss of FEN1K377me1 regulation as cells progress through S phase.

The Flap endonuclease activity of FEN1 is critical for DNA replication and is highly active during the S phase of the cell cycle [31]. Different post-translational modifications (PTM) including phosphorylation, and arginine methylation regulate FEN1 protein levels and activity at various stages of cell cycle [36, 37]. These known cell cycle dependent modifications prompted us to test whether K377me1 is also regulated during cell cycle progression. To test this hypothesis, we arrested cells at G_1_/S using HU. The synchronized cells were released from HU arrest and lysates collected at different time points and immunoblotted with the anti-FEN1K377me1 antibody. FEN1K377me1 increased as cells progressed through S phase and decreased, as they exited S phase (Figure 3A) and this up-regulation was SET7-dependent (Figure 3B). SET7 protein levels were not significantly regulated during S phase progression (Figure 3A, 3B). Cyclin E immunoblotting was used as a marker of cell cycle progression and tubulin as a loading control (Figure 3A, 3B). Propidium iodide analysis was used to monitor cell cycle progression (Figure 3A).

### FEN1 K377R mutant is sensitive to replicative stress following hydroxyurea treatment

**Figure 4:**
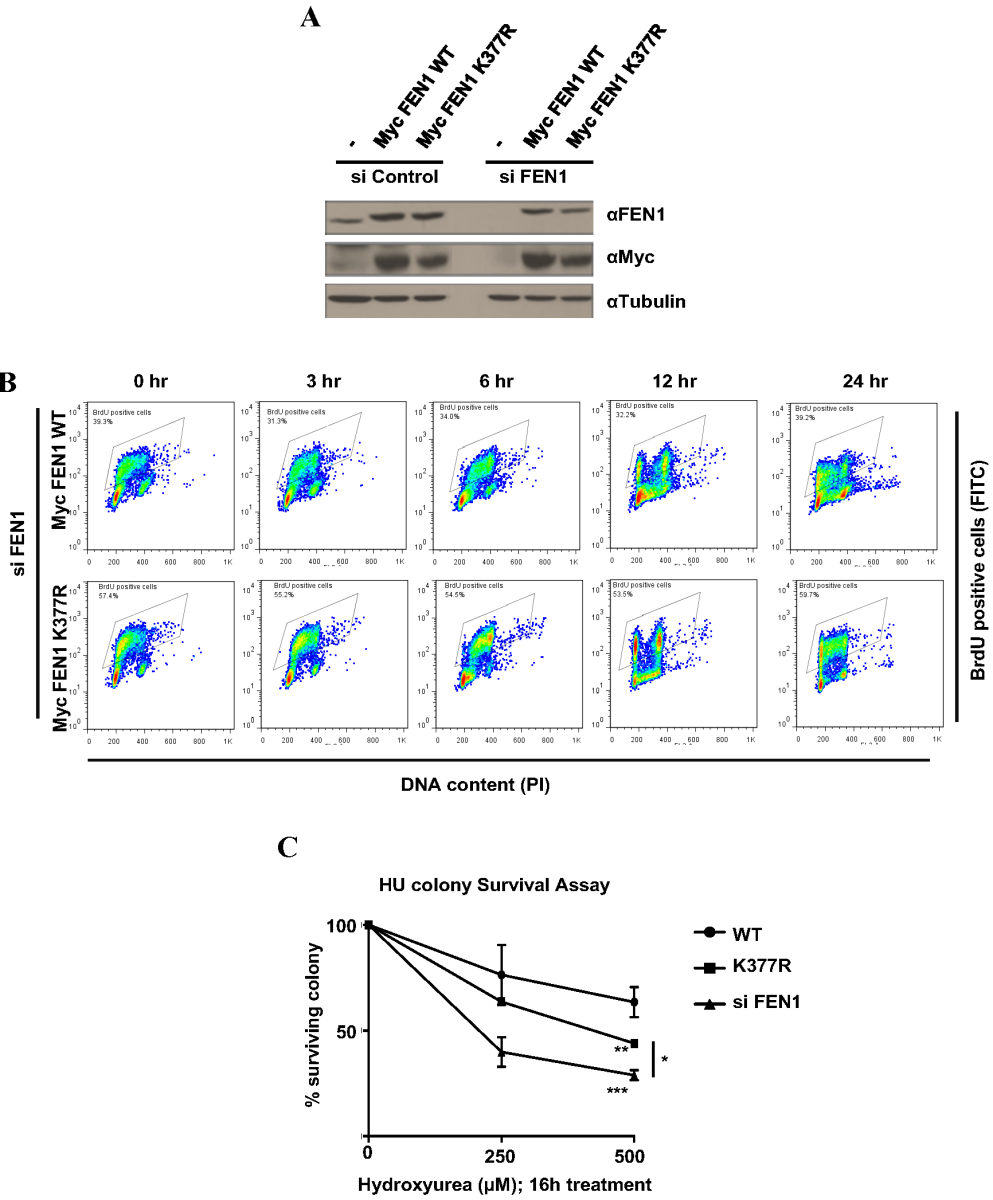
FEN1 K377 methylation is essential for cell survival to replicative stress **A.** U2OS cells stably expressing siRNA resistant Myc FEN1 WT and the K377R mutant were generated. Western blots showing the expression of Myc FEN1 WT and K377R mutant and the knockdown of endogenous FEN1. **B.** BrdU pulse chase to monitor cell cycle progression. U2OS cells stably expressing Myc FEN1 WT and K377R mutant were pulsed with BrdU for 30 min and were analyzed at different times as it progressed through cell cycle. **C.** Cells were treated with indicated doses of HU for 16 h. Survival cell colonies were counted. The error bars represent SD, (*n* = 2). Significance was measured by One way Anova followed by post hoc comparison using Bonferroni’s multiple comparison test. (*p < 0.05, **p < 0.005, ***p < 0.0005)

To understand the functions of FEN1 K377 methylation *in vivo*, we generated U2OS cell lines stably expressing siRNA resistant Myc-FEN1 WT and K377R mutant. Stable expression of the Myc constructs and the knockdown of endogenous FEN1 was confirmed by immunoblotting (Figure 4A). U2OS cells stably expressing Myc FEN1 WT and K377R mutant had similar cell cycle progression in normal conditions (Figure 4B). FEN1 had been previously reported to play a role in the resolution of stalled replication forks induced by camptothecin and UV treatment [38]. Since we observed FEN1K377me1 to be upregulated during S phase progression following HU treatment, we investigated whether cells stably expressing exogenous the FEN1K377R mutant was sensitive to replicative stress by colony formation assay. Following the knockdown of endogenous FEN1, cells expressing Myc FEN1 WT and K377R mutant were treated with either 250 or 500 µM HU. FEN1 K377R mutant cells were more sensitive to HU compared to cells expressing WT FEN1 (Figure 4C).

### SET7 methylation does not influence Flap endonuclease activity of FEN1

**Figure 5:**
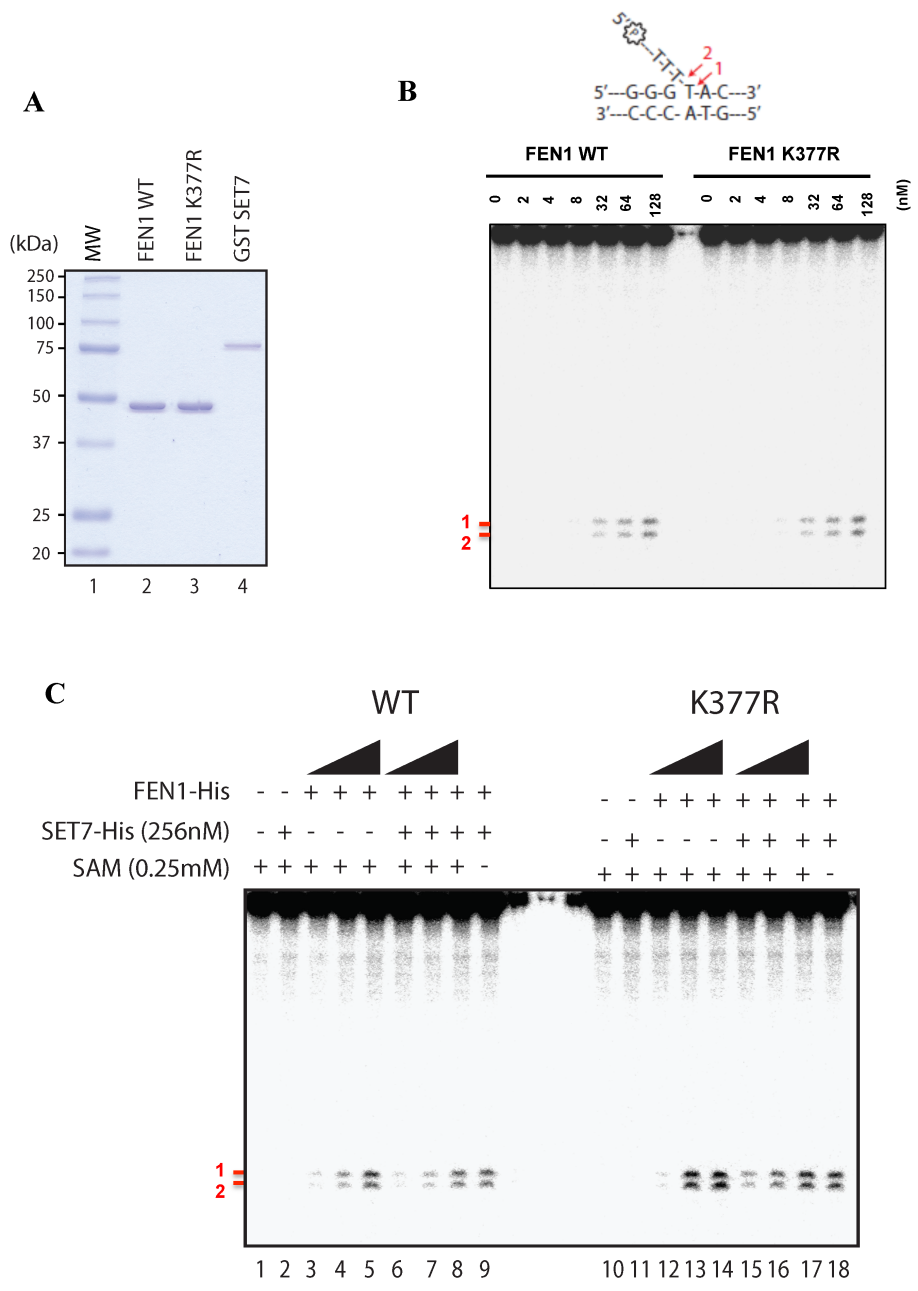
Flap endonuclease activity of FEN1 WT and K377R **A.** Coomassie stained SDS-PAGE gel of the purified proteins (250ng) used in Figure 4B. MW represents the molecular weight markers in kDa. **B.** Endonuclease assays were performed with increasing amount of FEN1 WT or FEN1 K377R and 200nM of FEN1 substrate. Products were visualized using a 10% denaturing gel and corresponding to bands denoted by # 1 and # 2 in red. Scheme of the FEN1 substrate is shown on the top of the gel. **C.** Methylation assay was achieved on FEN1 WT or FEN K377R (at 128, 256, 512 nM) using SET7 WT followed by endonuclease assays using 200 nM of FEN1 substrate.

We next investigated whether lysine methylation of FEN1 affected its Flap activity. We purified human SET7, FEN1 and FEN1K377R proteins from *E*. *coli* to homogeneity (Figure 5A). Using a radiolabeled DNA Flap structure, we showed that FEN1 could incise the 15-nucleotide DNA at two major sites (Figure 5B), as reported previously [39]. However, the FEN1K377R protein retained wild type activity (Figure 5B). We then used our assay in a methylation context. Briefly, we used the same conditions as before to methylate FEN1 wild type (WT) or FEN1 K377R by SET7 and analyzed the Flap activity using the FEN1 DNA substrate. Once again, the Flap activity was like wild type, suggesting that methylation of K377 did not affect activity (Figure 5C). As we aimed to understand the impact of SET7 methylation on the FEN1 activity, and that we previously showed a remaining level of methylation by SET7 for FEN1 K377R (Figure 1D - lane 7). We hence speculated that this remaining methylation could bias the role of SET7 methylation on K377R. We hence designed a polymutant of methylation potential sites (K354, K356, K365, K366, K369 and K377 replaced to arginines) named FEN1KRR. To gain insight in our assay, we also designed a well-known dead mutant SET7 (H297A) [40]. We purified all the proteins to homogeneity (Supplementary Figure 3A). We showed that the anti-FEN1K377me1 did not recognize the mutant FEN1KRR after methylation, and that SET7 H297A was effectively deprived of methylation activity (Supplementary Figure 3B). We show that the SET7 methylation of FEN1 did not have any consequence on its Flap activity and we noticed that mutants were more proficient in producing Flap nuclease products (Supplementary Figure 3C). This was likely due to an increasing DNA binding of mutant compare to FEN1 WT (Supplementary Figure 4A-C). Moreover, the mutant FEN1KRR was still able to interact with SET7, confirming it did not modify the binding activity of SET7 (Supplementary Figure 5). These results show that the SET7 methylation of FEN1 does not impact its Flap activity, suggesting another role of this post-translational modification.

## DISCUSSION

SET7 plays multiple roles in the DNA damage response (DDR) by methylating key cell cycle regulators such as p53, RB and E2F-1 [25-27]. To further explore the functions of SET7 in the DDR pathway, we screened a peptide SPOT array to identify potential new substrates of SET7 in DDR and repair. Many DDR proteins were identified to be methylated by SET7 *in vitro*. FEN1, an important structure specific endonuclease involved in the processing of FLAP structures that arise from Okazaki fragment maturation and was chosen for further functional characterisation of SET7 methylation. We identified SET7 to methylate FEN1 at K377 *in vitro* and *in vivo*. The cellular levels of K377me1 increased as cells progressed through S phase in a SET7-dependent manner. Moreover, SET7 methylation at K377 was found to be essential for cellular response to replicative stress, as cells expressing K377R mutant of FEN1 were sensitive to HU treatment.

SET7 was first implicated in the DDR, when it was shown to target p53 for methylation [25]. p53 methylation by SET7 following DNA damage treatment induces acetylation at K382 that leads to increased transactivation of p53 [28]. SET7 also activates p53 indirectly by disrupting p53-SIRT1 interaction that blocks deacetylation and repression of p53 [41]. However, recent studies on SET7 knockout mice have raised doubts on the functional role of SET7 methylation of p53 [42, 43]. SET7 methylation is also known to regulate cell cycle and apoptosis functions mediated by RB and E2F-1, respectively [27, 44]. In addition, Ivanov and coworkers, had earlier observed an increase in SET7 methyltransferase activity post-treatment with DNA damaging agent adriamycin [28]. In support of this observation, SET7 methylation of SIRT1, SUV39H1 and RB increases post-DNA damage [41, 44]. These reports define SET7 as a transducer of DNA damage signal and suggest SET7 could be involved in the methylation of several unidentified targets in the DDR pathway. Our peptide array screen identified many potential new substrates of SET7 in the DDR (Supplementary Table 2).

FEN1 is an important enzyme for the maintenance of genomic stability owing to its critical function in DNA replication and repair [29, 30]. We have identified SET7 as a new regulator of FEN1 functions in a cell cycle dependent manner. FEN1K377me1 was upregulated as cells progressed through S phase and decreased as they exited S phase. Interestingly, SET7 was previously reported to methylate DNA methyltransferase 1 (DNMT1) during S phase. Similar to FEN1K377me1, SET7 methylation of DNMT1 at K142 increases as cells progressed through S phase. A role for SET7 in S phase progression is further supported by the recent findings that SET7 coactivates E2F-1 dependent transcription of CCNE gene and SET7 knockdown arrests cells at G_1_/S and fail to transit through S phase [45]. However, methylation of FEN1 on K377 did not have any impact on its flap-activity neither on the interaction of important partners (such as BLM, PCNA and DNA2 (Supplementary Figure 6). Further work is required to identify the consequences FEN1 lysine methylation by SET7 function.

In sum, we identify FEN1 as an *in vivo* substrate of SET7 methylation and define K377 as the methylation site and is required for the cellular response to replicative stress.

## EXPERIMENTAL PROCEDURES

### Cells and antibodies

U2OS cells were from the American Type Culture Collection. αFEN1K377me1 antibody was generated in rabbits immunized with the FEN1peptide (368-380) carrying a monomethyl at K377. Rabbit anti-SET7 (C24B1) was purchased from Cell Signalling, rabbit anti-FEN1 (ab2619) was purchased from Abcam, anti-myc and anti-tubulin antibodies were purchased from Sigma-Aldrich. Mouse anti-GFP antibody was purchased from Roche, mouse anti-CyclinE, clone HE12 was purchased from Millipore.

### DNA constructs

Myc-FEN1 WT and K377R mutant were amplified using the primers

5′-GGGGGATCCCCACCATGGCAGAAACTCATCTCTGAAGAGGATCTGGGAATTCAAGGCCTGGCCAAACTAATTGC - 3′ Myc FEN1 forward; 5′-GGGCTCGAGTTATTTTCCCCTTTTAAACTTCCCTG -3′ reverse primer WT; 5′-GGGCTCGAGTTATTTTCCCCTTCTAAACTTCCCTG-3′ reverse primer K377R and cloned in at the *Bam*HI and *Xho*I sites in pcDNA3.1

FEN1 KRR mutant was generated byPCR amplifction of a gblock FEN1 template with K-R mutations of K354, K356, K365, K366, K369 and K377 and flanking *Sal*I and *Not*I restriction sites synthesized by IDT. The amplified fragment was digested with *Sal*I and *Not*I and sub-cloned in pGEX6P1. GST-SET7 was obtained from Dr. Or Gozani (Stanford University).

### Peptides

The peptide arrays were synthesized as previously described [46]. FEN1 WT: Biotin-AKTGAAGKFKRGK- ; FEN1 K369: Biotin-AKTGAAGRFRRGR-; FEN1 K375: Biotin-ARTGAAGKFRRGR-; FEN1 K377: Biotin-ARTGAAGRFKRGR-; FEN1 K380: Biotin-ARTGAAGRFRRGK; FEN1 K377me1: Biotin-AKTGAAGKFK*RGK; FEN1 K377me2: Biotin-AKTGAAGKFK**RGK; FEN1 K377me3: Biotin-AKTGAAGKFK***RGK; FEN1 (346-364): Biotin-VTGSLSSAKRKEPEPKGST; H3 (1-20): Biotin-ARTKQTARKSTGGKAPRKQL; H3K4me1: Biotin-ARTK*QTARKSTGGKAPRKQL; H3K9me1: Biotin-ARTKQTARK*STGGKAPRKQL; H3K36me1: Biotin-LATKAARKSAPATGGVK*KPH; H4K20me1: Biotin-GKGGAKRHRK*VLRDNIQGIT; p53 (360-380): Biotin-GSRAHSSHIKSKKGQSTSRH; H4 (10-30): Biotin-GKGGAKRHRKVLRDNIQGIT.

### Flashplate SET7 assay

The biotinylated peptides (330pmol in 50µl ddH_2_0) were allowed to bind to the streptavidin coated Flashplate (Perkin Elmer) overnight at 4°C. The peptides were then removed by suction and the Flashplate rinsed 4 times with PBS (0.05% Tween-20) and twice with ddH_2_O. The *in vitro* methylation reaction was then carried out using 1 µg of SET7 and 0.5 µl ^3^H-SAM in 50 µl reaction and incubated at 37°C for 2 to 4 h. The lysine methylation was measured by ^3^H scintillation counting on a Perkin Elmer Wallac MicroBeta.

### Cell lysis, immunoprecipitation and immunoblotting

Myc tagged FEN1 was expressed in U2OS cells using Lipofectamine 2000 (Invitrogen) as per manufacturer’s instructions. After 24 to 48 h, cells were lysed with cell lysis buffer (20mM Tris pH 7.4, 150mM NaCl, 1mM EDTA, 1mM EGTA, 1% Triton X-100). For immunoprecipitations, cell lysates were incubated with the primary antibody for 1 h. Then 25 µl of 50% protein A-Sepharose slurry was added and incubated at 4°C for 45 min with constant end-over-end mixing. The beads were then washed 2X with cell lysis buffer and once with PBS. The samples were then boiled with 25 µl of 2X SDS PAGE sample buffer, resolved in SDS polyacrylamide gels, transferred to nitrocellulose membranes and subjected to immunoblotting.

### siRNA transfections

Small interfering RNAs (siRNAs; Dharmacon Inc.) were transfected using Lipofectamine RNAi MAX (Invitrogen) as per the manufacturer’s protocol. The final concentration of the siRNA was 40 nM and the cells were lysed 72 h post-transfection. For SET7 siRNA, SMARTpool were purchased from Dharmacon Inc. For FEN1, the sequence of the siRNA used was 5′-GUU CUC UGA GGA GCG AAU C-3′. Luciferase siRNA was used as control.

### GST pull-down assays

GST, GST-SET7, FEN1 WT and FEN1 KRR were preincubated at 37°C during 15 min in GST buffer (20mM HEPES KOH pH 7.9, 150 mM KCl, 0.2 mM EDTA, 10% glycerol, 0.2% NP40, 0.5 mM DTT and protease inhibitor cocktail) complemented with 1mg/ml bovine serum albumin. Complexes were then incubated with gluthatione Sepharose beads (GE Healthcare) at 4°C for 1h. Complexes were washed four times with GST buffer without bovine serum albumin. Proteins were visualized by immunoblotting using the indicated antibodies.

### GST purification

GST constructs transformed into BL21 (DE3) bacterial strain were used to start 500 ml overnight cultures in 2x YT broth culture media supplemented with ampicillin (50μg/mL). The following day, when the cultures obtained an (O.D.600nm 0.5-0.7), GST expression was induced for 4h with 0.1mM IPTG (Invitrogen). The cells were collected and suspended in 10mL lysis buffer (50mM Tris-Cl, pH7.5, 150mM NaCl, 0.1% NP-40, 10% glycerol, supplemented with EDTA-free complete protease cocktail (Roche), freshly added) and sonicated. The lysates were then cleared by centrifugation and incubated with glutathione-agarose (Sigma) for 1h at 4°C, washed with lysis buffer 5x and the bound GST proteins were eluted with 10mM reduced glutathione.

### Mass spectrometry of *in vitro* methylated FEN1 peptide 368-380

1µg of the FEN1 peptide 368-380 was incubated with 1µg of GST-SET7 or without GST-SET7 (control) and 0.1 mM S-adenosyl-methionine (SAM) (AdoMet; Sigma) in a reaction buffer containing 50 mM Tris-HCl (pH 8.0), 10% glycerol, 20 mM KCl, 5 mM MgCl_2_ in a final reaction volume of 25 µl. The reaction was incubated at 37°C for 2h. Following incubation, the samples were subjected to LC-MS/MS analysis.

### Homology modeling

The homology model of *Homo sapiens* SET7/9 was generated using the SET7/9-H3-AdoHCy ternary complex (Protein Data Bank structure accession no. 1o9s) as a template [34]. The histone H3 peptide was manually mutated, using Coot, to the corresponding Fen1 (residues 375 to 380 methylation site. Manual adjustments to the model were performed by using Coot [47]. The final model encompasses residues 108 to 366 of SET7/9, the 6-residue Fen1 peptide, and AdoHcy. The stereochemistry of the SET7/9-Fen1-AdoHCy model was validated by using Molprobity, which verified that nonglycine residues are absent from the disallowed regions of the Ramachandran plot. Structural figures were rendered in PyMOL (http://pymol.sourceforge.net/).

### Colony assay

A total of 200 to 1000 cells were plated on 10-cm dishes in triplicates and treated with various concentrations of HU as indicated in the figure for 24h. Following 24h of treatment, the cells were washed twice with phosphate-buffered saline (PBS). Colonies were allowed to grow for 2 weeks. At the end of 2 weeks, the colonies were fixed for 10 minutes with 4% paraformaldehyde and stained with 0.1% crystal violet for 30 min. Stained colonies were counted and the percentage surviving colony was determined by dividing the average number of colonies in each treatment to the average number of colonies in the control treated plates.

### *In vitro* methylation

1 µg GST FEN1 was incubated with 1 µg of GST-SET7 and 0.55 µCi ^3^H-SAM in reaction buffer containing 50 mM Tris-HCl (pH 8.0), 10% glycerol, 20 mM KCl, 5 mM MgCl_2_ in a final reaction volume of 25 µl. The reaction was incubated at 37°C for 2-4 h. Reaction was stopped by adding 25 µl of 2X SDS PAGE sample buffer, followed by heating at 100°C for 10 min. The reaction mixtures were then subjected to electrophoresis on SDS-polyacrylamide gels and stained with Coomassie blue. The destained gels were soaked in EN^3^HANCE (Perkin Elmer) according to manufacturer’s instructions and visualised by fluorography.

### Cell synchronization and flow cytometry

U2OS cells were synchronized with 1mM HU (Sigma) for 12 to 16 h. To release arrested cells, they were washed three times with 1 X PBS and resubstituted with fresh media. For flow cytometry (FACS) analysis, U2OS cells were collected and fixed with 70% ethanol at 4°C overnight. Cells were then washed with PBS and stained with 10 µg/ml propidium iodide at room temperature for 2 h. The samples were analyzed by FACS.

### Preparation of flap substrates and assay

Oligonucleotide sequences [39] #1 (5’-AAG CCA CTG CAG GTC GAC TCT AGA GGA TCT CCG GG-3’), #2 (5’-GCT CTT CGA GAA TTT TAC CGA GCT CGA ATT CAC TGG CCG TCG TTT TAC AAC GTA-3’), and #3 (5’-TAC GTT GTA AAA CGA CGG CCA GTG AAT TCG AGC TCG GTA CCC GGA GAT CCT CTA GAG TCG ACC TGC AGT GGC TT-3’) were used. Oligonucleotide #2 was 5’ labeled with ^32^P using T4 polynucleotide kinase and ^32^P-β-ATP (NEB) during 1h. The reaction was divided in equal amount to allow controls of the annealing (single strand, double strand and triple strand). Oligonucleotides #1 and #2 were then added in a specific manner, and annealing was processed in a PCR machine within 48 min, slowly cooling from 94°C to 12°C (0.03°C/s). Each product was purified using acrylamide gel. The tripartite product was named FEN1 substrate.

Reactions containing 200 nM of ^32^P tripartite probe were incubated with indicated concentration of proteins in 10 µl of FEN1 buffer (50 mM Tris HCl pH 8.5, 20 mM KCl, 1 mM DTT and 5 mM MgCl_2_) for 20 min at 37°C. Reactions were stopped by adding 10 µl of denaturing dye (95% formamide with 20 mM EDTA). Samples were boiled for 5 min at 95°C. Half of the reaction was load on a 10% denaturing gel at 75W for 1h, dried on a Whatmann paper and visualized using a Phosphoimager.

## SUPPLEMENTARY MATERIALS FIGURES AND TABLES






