# Supplementary material for: Lysine methylation of FEN1 by SET7 is essential for its cellular response to replicative stress
Source: Oncotarget. 2017 May 22;8(39):64918–31. doi: 10.18632/oncotarget.18070 (PMC5630301; doi:10.18632/oncotarget.18070)
Supplement: Supplementary file 1 [file oncotarget-08-64918-s001.pdf]

Lysine methylation of FEN1 by SET7 is essential for its cellular response to replicative stress

Supplementary Material

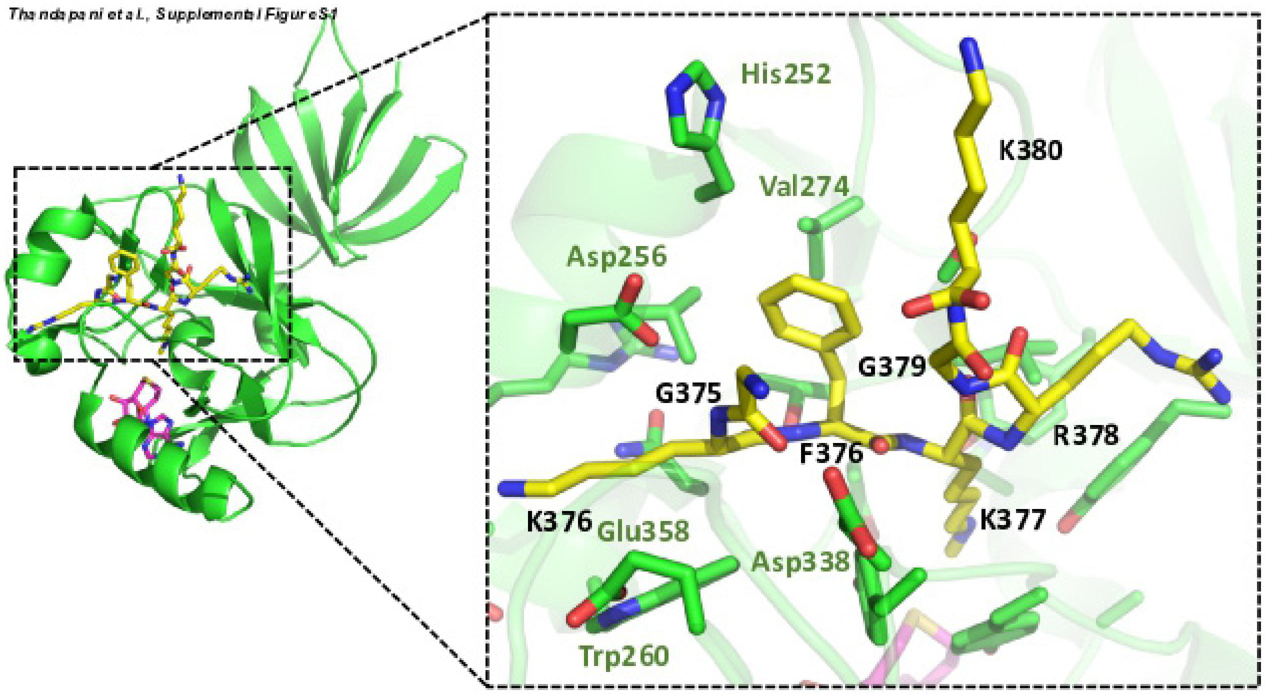

Supplementary Figure S1. Homology model of SET7/9 catalytic domain in complex with Fen1. A) Ribbon representation of the secondary structure of the SET7/9 catalytic domain illustrating the SET domain (green) and Fen1 peptide spanning residues 375 to 380 (yellow carbon atoms) and the product AdoHcy (magenta carbons), are docked in the active site. B) Fen1 peptide binding cleft of SET7/9. Residues comprising the enzyme's peptide binding cleft are shown with green carbon atoms. AdoHcy and Fen1 are depicted as described for panel A.

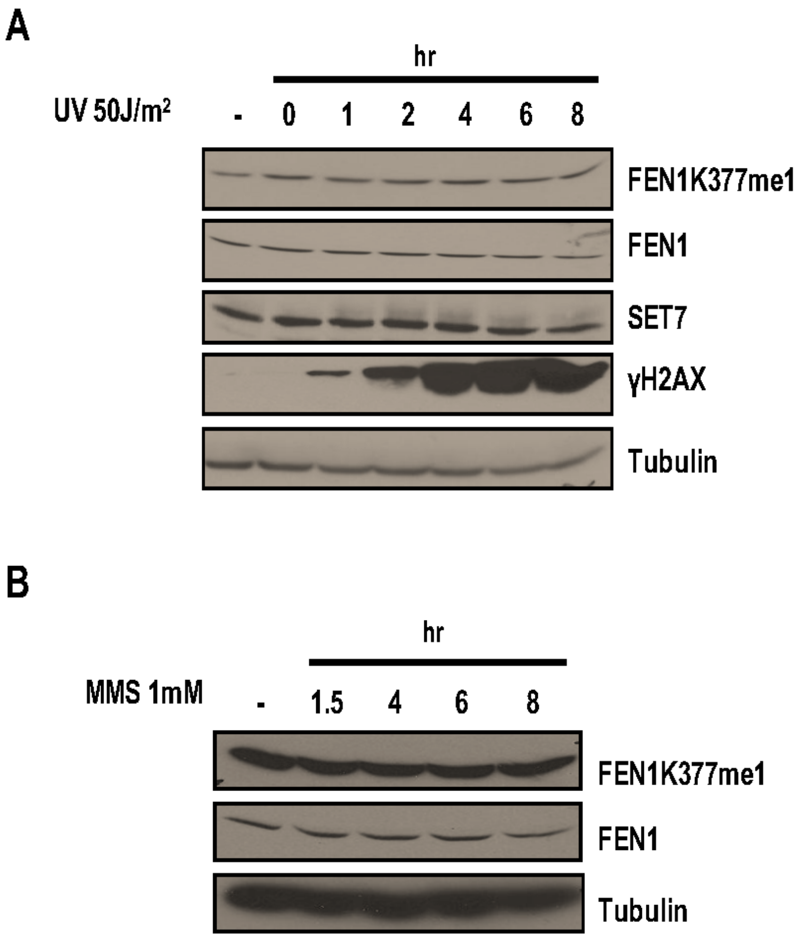

Supplementary Figure S2. FEN1K377me1 is not regulated in response to DNA damage treatment. U2OS cells were treated with 50 J/m_2 UVC A) or 1 mM MMS B). Cells were collected at various time points post-treatment and lysates were immunoblotted with anti-FEN1K377me1, FEN1, SET7, γH2AX and Tubulin antibodies.

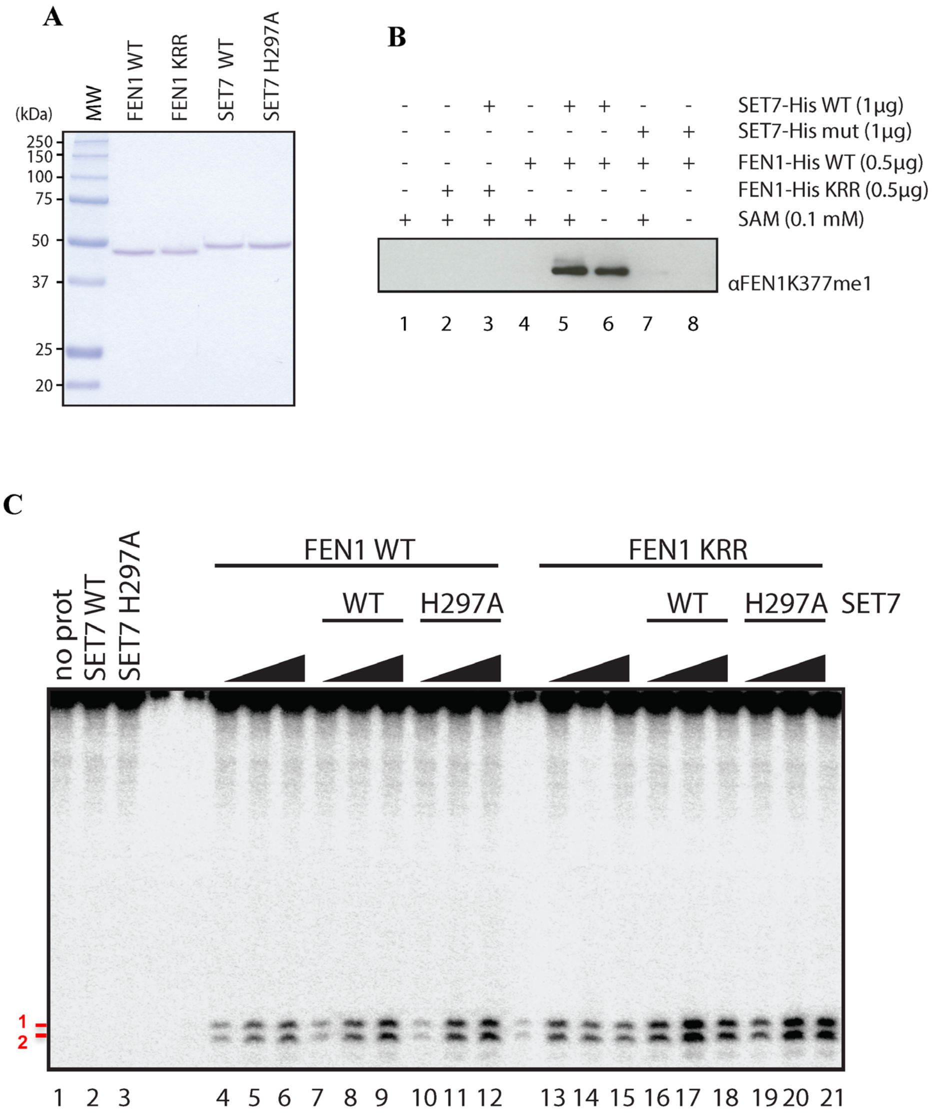

Supplementary Figure S3. FEN1 methylation using purified proteins and flap endonuclease assays. A) SDS-PAGE of the Coomassie Blue stained proteins (250 ng). MW represents molecular weight markers in kDa. B) In vitro methylation of FEN1 WT and FEN1 KRR with SET7 or SET7 H297A. C) Methylation assay was achieved on FEN1 WT or FEN KRR (at 128, 256, 512 nM) using SET7 WT or SET7 H297A followed by endonuclease assays using 200nM of FEN1 substrate. Products were visualized using a 10% denaturing gel and corresponding to the bands denoted by the # 1 and # 2 in red.

A

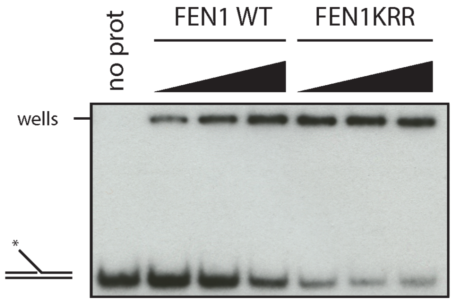

B

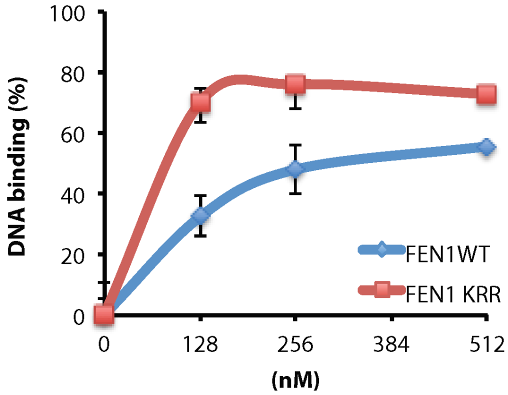

C

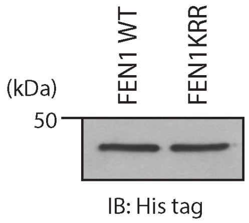

Supplementary Figure S4. DNA binding activity of FEN1 WT compared to FEN1 KRR. A) Electophoretic mobility shift assays (EMSAs) were performed with 128, 256 or 512 nM of FEN1 WT or FEN1 KRR. B) Quantification of the result from 3 independent experiments. C) Western blot against HIS-FEN1 WT and HIS-FEN1 KRR show the equal amount of protein used in the DNA binding assay.

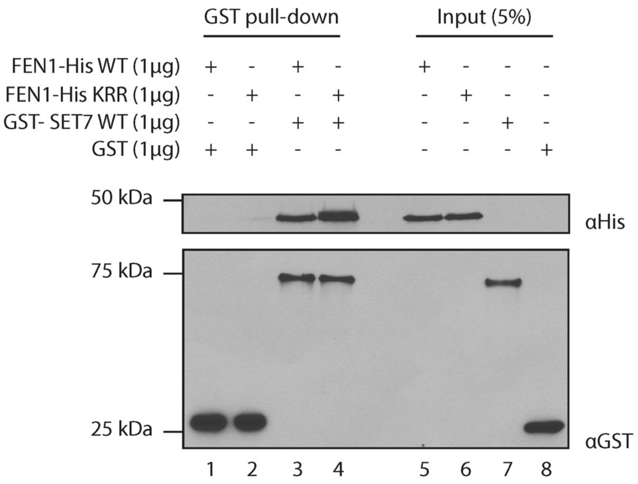

Supplementary Figure S5: Direct interaction between FEN1 and SET7. GST pull-down assays of GST-SET7 in the presence of FEN1WT or FEN1 KRR. Proteins were detected by Western blot using anti-GST or anti-HIS antibodies.

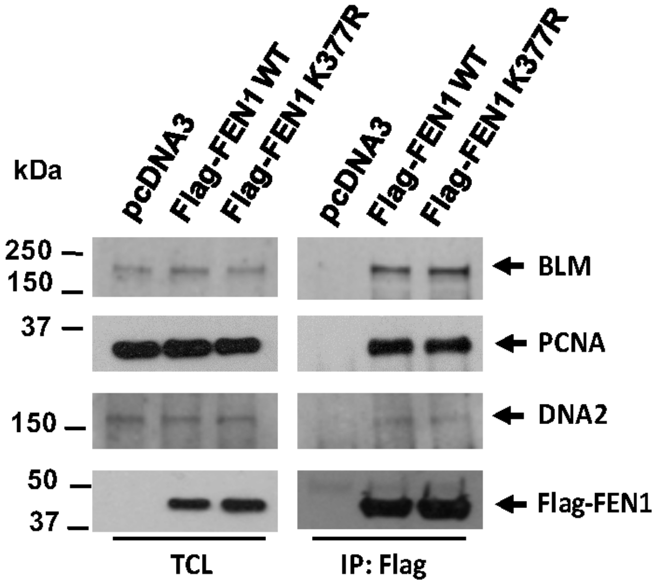

Supplementary Figure Figure S6. The K377R mutation has no effect on FEN1 association with BLM, PCNA or DNA2. HEK293 cells were transfected with pcDNA3 empty vector, Flag-FEN1 wildtype and Flag-FEN1 K377R mutant, respectively. Three days after transfection, the whole cell lysates were subjected to IP with Flag-agarose beads and WB with anti-BLM, PCNA, DNA2 and Flag antibodies
